# Entropy and Random Walk Trails Water Confinement and Non-Thermal Equilibrium in Photon-Induced Nanocavities

**DOI:** 10.3390/nano10061101

**Published:** 2020-06-02

**Authors:** Vassilios Gavriil, Margarita Chatzichristidi, Dimitrios Christofilos, Gerasimos A. Kourouklis, Zoe Kollia, Evangelos Bakalis, Alkiviadis-Constantinos Cefalas, Evangelia Sarantopoulou

**Affiliations:** 1 National Hellenic Research Foundation, Theoretical and Physical Chemistry Institute, 48 Vassileos Constantinou Avenue, 11635 Athens, Greece; vgavriil@eie.gr (V.G.); zkollia@eie.gr (Z.K.); evangelos.bakalis2@unibo.it (E.B.); ccefalas@eie.gr (A.-C.C.); 2 School of Chemical Engineering and Physics Laboratory, Faculty of Engineering, Aristotle University of Thessaloniki, University Campus, 54124 Thessaloniki, Greece; christof@eng.auth.gr (D.C.); gak@auth.gr (G.A.K.); 3 Department of Chemistry, Laboratory of Industrial Chemistry, Panepistimiopolis Zografou, National and Kapodistrian University of Athens, 15771 Athens, Greece; mchatzi@chem.uoa.gr; 4 Dipartimento di Chimica “G. Giamician” University di Bologna, Via F. Selmi 2, 40126 Bologna, Italy

**Keywords:** nanocavities, non-thermal equilibrium, water, entropy, nanothermodynamics, nanoindentation, AFM, electric dipole interactions, VUV irradiation, random walk

## Abstract

Molecules near surfaces are regularly trapped in small cavitations. Molecular confinement, especially water confinement, shows intriguing and unexpected behavior including surface entropy adjustment; nevertheless, observations of entropic variation during molecular confinement are scarce. An experimental assessment of the correlation between surface strain and entropy during molecular confinement in tiny crevices is difficult because strain variances fall in the nanometer scale. In this work, entropic variations during water confinement in 2D nano/micro cavitations were observed. Experimental results and random walk simulations of water molecules inside different size nanocavitations show that the mean escaping time of molecular water from nanocavities largely deviates from the mean collision time of water molecules near surfaces, crafted by 157 nm vacuum ultraviolet laser light on polyacrylamide matrixes. The mean escape time distribution of a few molecules indicates a non-thermal equilibrium state inside the cavity. The time differentiation inside and outside nanocavities reveals an additional state of ordered arrangements between nanocavities and molecular water ensembles of fixed molecular length near the surface. The configured number of microstates correctly counts for the experimental surface entropy deviation during molecular water confinement. The methodology has the potential to identify confined water molecules in nanocavities with life science importance.

## 1. Introduction

Confined molecular water in nanocavities shows intriguing and unexpected behavior. The dynamic evolution of confined molecular water swings between bulk response, molecular collective actions and interface binding reactions [1]. Translational and rotational motions of confined water point to different stretching dynamics from its bulk counterpart [2]. It is also known that confined water builds tight hydrogen-bonded (H-bonded) networks, and its flow response is diverging by orders of magnitude from macroscopic hydrodynamics [3]. Possible lack of H-bonding of water molecules in small volumes counts for de-wetting, cavity expulsion [4], water self-dissociation [5] and a diverging dielectric constant [6]. It is plausible; therefore, that diverging behaviors of the biological and geological evolution of molecular enclosures in small systems [7,8,9,10,11,12,13] also imply a nanothermodynamic approach [14,15]. 

The central element of any thermodynamic theory of small systems is based on the hypothesis that nanometer-sized configurations pullout an additional physical component to the free energy of the associated macroscopic system from interactions among nanostructure entities. Moreover, the confinement of a relatively large number of molecules in nanocavities, restraints the molecular degrees of freedom (translational, vibration or rotational), and finally the system evolves through different entropic states before equilibration. Most interesting, the confinement of a small number of molecules in a large number of distinguishable tiny spaces might well indicate a thermodynamic entropic collective behavior [13], space and time local heterogeneities, not-extensive fluctuations and intriguing surface-boundary effects. The reduction of the translational degrees of freedom of molecules in tiny spaces and the deviation of the molecular trapping time inside a cavity from the mean molecular collision time outside, highlight the presence of an entropic barrier that separates the molecular motions inside and outside the cavities. 

Today, both theoretical [14,15,16,17,18,19] and experimental advancements [20,21] gradually disclose the intriguing issues of thermodynamics of small systems, with major impacts on colloids, liquids, surfaces, interphases, chemical sensors, micro/nanofluidics, nanoporous media, proteins and DNA folding [10,22,23,24,25,26,27]. In cell biology, the presence of different nano-sized molecular scaffolds in the extracellular matrix environment implies a vast diversity of cellular activities and responses, including uncorrelated diverging drug delivery efficiencies [28].

Because thermodynamic potential variations and fluctuations allow for volume and surface stressing, any experimental verification of local volume and surface stress might well point to entropic fluctuations during molecular confinement [13,29]. Commonly, bulk and surface stressing go along with self-assembled structures, translational symmetry breaking, non-linearity, bifurcations, chaos, instability and morphological and shape nano configurations [30,31]. In the non-equilibrium state, rapidly changing thermodynamic potentials across phase boundaries usually force tiny systems to pass from different morphological progressions and physical states by tracing minimum energy and maximum entropy production pathways. This universal principle appears everywhere in Nature; from self-assembled bio and macromolecular structures and folding of large protein molecules [32] to nano/micro flower-like artificial structures [33,34].

The confinement of molecules within nano-size cavitations, usually on the surface of a matrix, is linked to system’s entropy diversity before and after trapping [13,27,35,36]. It is also known that for the same translational entropy, any confined molecular state attains a small variation of its rotational entropy compared to the non-confined molecular state. Likewise, rotational restriction affects surface molecular bonding and sorption/desorption kinetics [35]. Specific response of nanoentropic potentials from molecular confinement within photon-induced nanocavitations in PDMS matrixes underlines an inherent correlation between internal stressing and 2D entropy diversion [13]. 

Commonly, photon-processing of surfaces reconfigures their physicochemical properties, including thermodynamic potentials [37,38,39,40]. Irradiation of a polymeric matrix with vacuum ultraviolet (VUV) light in the spectral range from 110 to 180 nm entails an extensive modification of topological and thus of physical features, because of bond breaking and formation of new bonds. Any 2D topological transform is accompanied by a diversion of surface characteristics, such as porosity, sensing efficiency, chemical stability and extensive nanocavitation [41,42,43,44,45,46]. The adsorption of various molecules on 2D nanostructured surfaces [47,48,49], might well boost a plethora of surfactant effects along with molecular sensing [43,50], gas separation and storage [51,52,53,54], and also applications with particular emphasis on nanomedicine [55], bio-engineering [56,57] and drug delivery systems [58,59]. Among other polymeric matrixes, polyacrylamide (PAM) is a hydrophilic low toxic, biocompatible, water-soluble, synthetic linear or cross-linked molecule, modified accordingly for a wide range of applications, including oil recuperation, wastewater treatment, soil conditioner, cosmetics food and biomedical industries [60,61,62]. A diverging number of physical and chemical methods are currently applied to optimize the biocompatibility level of different polymers (e.g., PDMS, PET, PTFEMA, PEG), for biomedical applications, biosensors, tissue engineering and artificial organs [46,63,64]. Well established methods of surface functionalization through photon irradiation with UV, VUV and EUV (extreme ultraviolet) light sources and plasma treatment at various wavelengths and electron energies, aim to optimize chemical instability and surface modification for controlling a plethora of surface functionalities [65].

Today, several methods exist to improve the strength and the physicochemical properties of PAM matrixes by blending the matrix with chitosan, starch or other polymers [66]. While functionalization of pure PAM polymeric surfaces is mostly done via sunlight exposure at standard environmental conditions, a limited number of studies include plasma processing [67,68,69,70,71]. However, no data exist for VUV processing of PAM surfaces, preventing thus precise tailoring of PAM’s physicochemical surface characteristics (surface roughness, structure size, elasticity, chemical composition, etc.) and the formation of controlled micro/nanopatterns and cavitations for different applications [37,42,43,63,64].

The current work establishes the link between entropy variation and molecular water confinement in small nanocavities fabricated by 157 nm laser photons in polymeric PAM matrixes. The work follows a line of a rational evolution. First, the correlation between 157 nm molecular photodissociation (laser fluence or a number of laser pulses) and surface topological features, including nanocavitations, is established from fractal and surface analysis by using atomic force microscopy (AFM). Next, the correlation between surface strain and 157 nm molecular photodissociation is revealed by applying AFM nanoindentation (AFM-NI), contact angle (CA) wetting and white light reflection spectroscopy (WLRS). Random walk simulations of water molecules inside cavitations differentiate the escape time of confined molecular water and the mean collision time of water molecules near the PAM surface. The different time scales inside and outside the nanocavities point to an additional state of ordered arrangements between nanocavities and the molecular water ensembles of fixed molecular length near the surface. The configured number of microstates properly counts for the experimental surface entropy deviation during molecular water confinement, in agreement with the experimental results. Finally, the mean time distribution for a small number of water molecules for different runs reveals a non-equilibrium state inside tiny cavities. The experimental method has the potential to identify confined water molecules in nanocavities via entropy variation. The proposed roadmap of analysis may be used in applications related to life science.

## 2. Materials and Methods 

### 2.1. Materials

PAM (typical M_n_ = 150 K, M_w_ 400 K) purchased from Sigma-Aldrich (St. Louis, MO, USA) used to prepare solution 5% *w*/*w* in water. Thin layers (426 ± 1 nm) on Si wafer substrates were made by spin-coating for 60 s at 2500 rpm, and finally, cured at 110 °C for 15 min at a temperature rate of 0.37 °C s^−1^ and then left to cool at room temperature. WLRS measures the thickness of PAM films coated on Si wafers. 

### 2.2. 157 nm Laser

PAM layers irradiated with a high power pulse discharged molecular fluorine laser at 157 nm (Lambda Physik 250 (LPF^TM^ 200), Lambda Physik AG (Coherent), Göttingen, Germany), under continuous nitrogen flow (99.999%) at 10^5^ Pa and room temperature. The layers were mounted into a computer-controlled *X-Y-Z-θ* translation-rotation motorized stage, placed inside a 316 stainless-steel chamber. The laser temporal pulse duration at FWHM, the energy of an unfocused laser beam per laser pulse, the photon fluence per laser pulse and laser repetition rate were set up at 15 ns, 28 mJ, 250 Jm^−2^ and 10 Hz. For dipping the amount of oxygen inside the stainless-steel chamber, nitrogen purging of the chamber was applied for 10 min before the irradiating stage.

### 2.3. AFM Imaging and AFM-NI

An AFM system (*diInnova*, Veeco Instruments Inc. (SPM Bruker), Santa Barbara, CA, USA) used for surface imaging of exposed/non exposed areas and the AFM-NI measurements. The imaging carried out in a tapping mode at a scanning rate of 0.5 Hz, using phosphorus-(n)-doped silicon cantilever (MPP-11123-10), having a spring constant of 40 nN nm^−1^ and tip radius of 8 nm, operating at a resonance frequency of 300 kHz at ambient conditions. The surface parameters of the samples were also evaluated.

The force versus distance (F-D) response from ten different points on each non-exposed and exposed areas was also recorded with the same cantilever. The elastic modulus (Young’s modulus) was calculated using the SPIP force curve analysis software by fitting a Hertz model to the force-distance curve. The hysteresis between approach and retract curves were corrected by the same software. Calculations performed with a Poisson’s ratio value of 0.3 [72].

### 2.4. Fractal Analysis

The fractal characteristics of the exposed and non-exposed areas were quantified through the fractal dimensionality D*_f_* that describes the topology and the cavitation of a surface quantitatively. D*_f_* was derived from AFM images by four different algorithms, the cube counting, triangulation, variance and power spectrum methods, besides an algorithm provided by the AFM’s “lake pattern” software (diSPMLab Vr.5.01). A detailed description of the concept and the specific methodologies of the different algorithms can be found in [27]. The D*_f_* was calculated for the four different methods using “Gwyddion, SPM data visualisation and analysis tool” [73]. The D*_f_* calculated with the four different algorithms follow the same trend, despite small dimensionality divergences coming up from systematic errors, because of the different converging speed of the fractal analytical approaches.

### 2.5. Water Contact Angle (CA) 

The chemical modification of PAM surfaces following PAM surface laser irradiation was monitored by water CA surface measurements under ambient atmospheric conditions. Distilled water droplets with a volume of 0.5 μL were gently deposited onto the sample surface using a microsyringe. Water CAs on samples before and after irradiation and at different time intervals were measured using a CA measurement system (Digidrop, GBX, Romans sur Isere, Drôme, France) equipped with a CCD camera to capture lateral snapshots of a droplet deposited on top of the preselected area, suitable for both static and dynamic CA measurements. Droplet images captured at a speed of 50 frames/s. CA values were obtained via the Digidrop software analysis, approximating the tangent of the drop profile at the triple point (three-phase contact point). Three different CA measurements were taken from each sample at different sample positions to calculate the average values.

### 2.6. White Light Reflectance Spectroscopy (WLRS) 

The WLRS measurements were performed by an FR-Basic, ThetaMetrisis™ (ThetaMetrisis SA, Athens, Greece) equipped with a VIS–NIR spectrometer (Theta Metrisis SA, Athens, Greece) having 2048 pixels detector and optical resolution of 0.35 nm. The beam of the light source comes from a white light halogen lamp, with a uniquely designed stable power supply and soft-start circuit, ensuring stable operation over time that is necessary for long time duration experiments. Software controls the instrument, performing the data acquisition and film thickness calculations. The PAM films were spin-coated on native oxide Si wafers and SiO_2_ layer on the top with a thickness of 2–3 nm. 

### 2.7. Random Walk Model

The mean escape time of a water molecule confined in nanocavities was computed by applying different 3D random walk models with diverging numbers of water molecules, variable spherical size nanocavities, and entrance-escape hole sizes. Two different models of non-interactive and interactive water molecules inside the cavities were used. The first model, the non-interactive random walk model, uses molecular masses of zero volume and elastic collisions of the water molecule with the cavity wall and it records the sequence of positions of water molecules inside the spherical cavity until it gets back to the entrance-escape hole. The collision angle was varied randomly with a uniform distribution. The model calculates the total distance that molecules travel in the cavity before they escape from the entrance-escape hole. The mean escape time was calculated by considering that the molecule attains its kinetic energy after an elastic collision with the walls of the cavity. Therefore the kinetic energy transfer from the wall to the molecule should be equal with the thermal energy of the wall $\frac{3}{2}k_{B}T$, where $k_{B}$ is Boltzmann’s constant.

The escape time from the entrance-escape hole for a non-interactive water molecule in the cavity is given by the equation:

(1) $$t_{e}=\frac{\sum_{i=0}^{n}\left(\sqrt{2R^{2}(1-\sin\theta_{i+1}\sin\theta_{i}\cos\left(\varphi_{i+1}-\varphi_{i}\right)+\cos\theta_{i+1}\cos\theta_{i}}\;\right)-2R+\left(R_{0}+R_{n}\right)}{\sqrt{\frac{3k_{B}TN_{A}}{M_{H_{2}O}}}}$$

where *n* is the number of collisions in each run, *R* is the radius of the spherical cavity, $\left(R,\theta_{i},\varphi_{i}\right)$ is the position of the molecule in the *i*th collision. The entrance and the exit point in the cavity wall are given in spherical coordinates $\left(R_{0},\theta_{0},\varphi_{o}\right)$ and $\left(R_{0},\theta_{n},\varphi_{n}\right)$, accordingly, and $M_{H_{2}O}$ is the molecular mass of water.

The interactive random walk model records the sequence of positions of a specific molecule that enters a spherical cavity through the entrance-escape hole, alongside with the locations of a variable number of neighboring molecules trapped in the cavity, until it gets back to the entrance-escape hole. At first, because of non-thermal equilibrium between water molecules within the cavity, the molecules are placed inside the cavity in random positions with random velocities of uniform distribution between 0 and $\sqrt{\frac{3k_{B}TN_{A}}{M_{H_{2}O}}}\;m/s$. The position of each molecule was recorded every$\;10^{-14}s$. The collision of each water molecule with the cavity wall and its neighbouring molecules is considered to be elastic. The collision angle was varied randomly with a uniform distribution. Contrary to the non-interactive model of zero-size molecules, the interactive model uses a spherical molecular diameter of 0.3 nm.

For every pair of the cavity size and entrance-escape hole, the random walk was run 10^2^ times and the mean escape time was calculated. In addition, the mean-escape time distribution for different cavities and number of molecules was used to evaluate the thermodynamic state inside the cavity. The model was designed and run in MATLAB. 9.4.0.813654 (R2018a), The MathWorks Inc.; Natick, MA, USA.

## 3. Results

### 3.1. Surface Analysis

**Figure 1:**
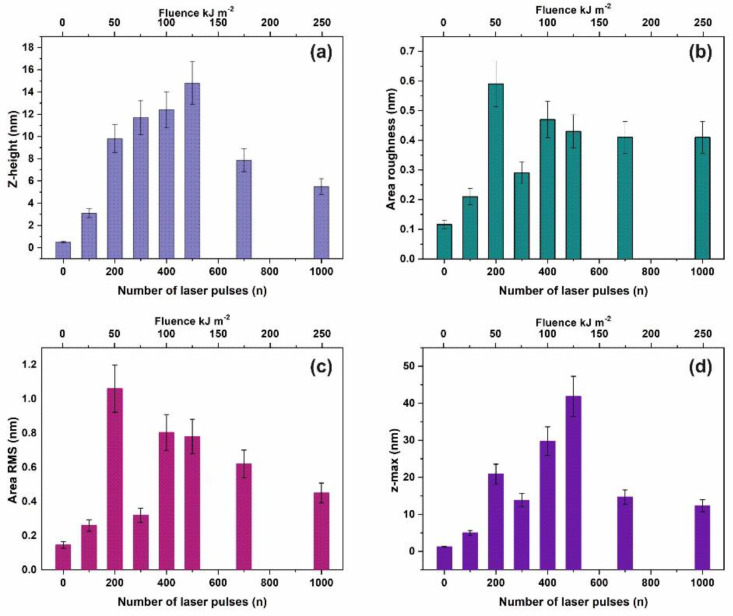
Surface parameters of irradiated polyacrylamide (PAM) layers for a 2 μm × 2 μm area: (**a**) Mean *z-*height; (**b**) area roughness (R_a_); (**c**) area RMS; (**d**) maximum range. The area roughness and area RMS parameters show an increment with laser pulses up to ~ 200 lp followed by a dip at 10^3^ lp.

**Figure 2:**
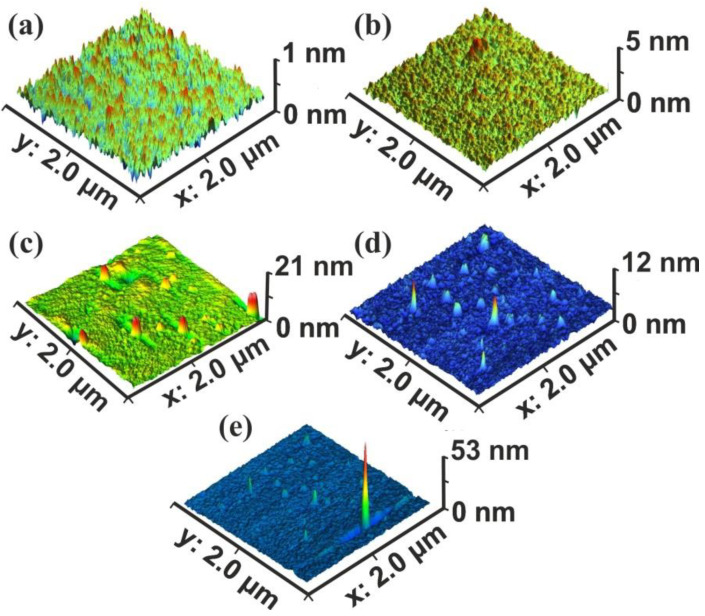
Atomic force microscopy (AFM) surface image of polyacrylamide (PAM) layers. Scan area 2 × 2 μm^2^, laser fluence 250 J m^−2^ per pulse: (**a**) non-irradiated PAM layer; (**b**) irradiated PAM layer with 100 laser pulses (lp), 25 kJ m^−*2*^; (**c**) 200 lp, 50 kJ m^−2^; (**d**) 10^3^ lp, 250 kJ m^−2^; (**e**) scan area 2.3 × 2.3 μm^2^, 10^3^ lp, 250 kJ m^−2^. The surface morphology is area size-dependent.

Commonly, four surface parameters, the surface roughness histogram, the area roughness, the area root mean square (RMS) and the maximum range characterize a surface and mean area values are plotted as functions of the laser pulse number or the laser fluence, Figure 1. The surface parameters are extracted from AFM images, Figure 2a–e. 

The surface roughness histogram, or average *z*-height, is the arithmetic mean defined as the sum of all height values divided by the number of data points $\left|Z\right|=\frac{1}{N}\overset{N}{\underset{i=1}{\sum }}Z_{i}$. Next, the *R_a_* (area roughness or roughness average) is the arithmetic mean of the height deviation from the image’s mean value,$\;R_{a}=\frac{1}{n}\overset{n}{\underset{i=1}{\sum }}\left|Z^{i}-\bar{Z}\right|$. The area RMS (*R_rms_*) is the value defined as the square root of the mean value of the squares of the distance of the points from the image mean value: $R_{rms}=\sqrt{\frac{1}{N}\overset{N}{\underset{i=1}{\sum }}{\left(Z-Z_{i}\right)}^{2}}$. Finally, the maximum range of *Z_max_* is defined as the maximum value of *z-*heights. The surface parameter values (*z*-height, area roughness, area RMS, and maximum range) of photon exposed areas were more considerable compared to the non-irradiated ones. However, because surface parameters are area size-dependent (Figure 2d,e), they are utilized only for a comparative qualitative evaluation of area modification under 157 nm laser irradiation.

### 3.2. Fractal Analysis of 157 nm Photon Processed PAM Polymeric Matrixes

**Figure 3:**
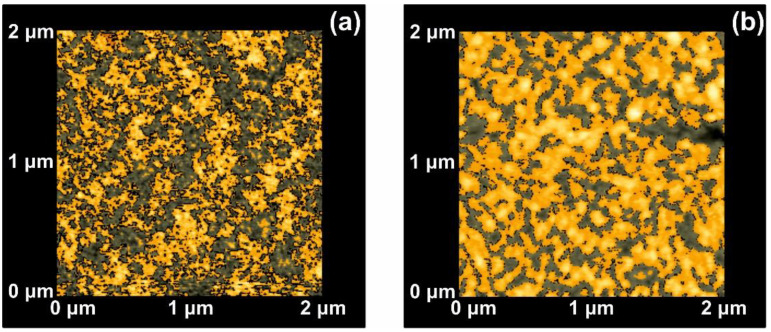
Atomic force microscopy (AFM) image of ‘‘lake’’ (grey) and ‘‘island’’ (orange) for a fractal area of 2 × 2 μm*^2^*: (**a**) Non-irradiated area; (**b**) irradiated area with 10^3^ laser pulses.

Because of statistical self-similarity between matrix space topology during a scaling-down route, there is a strong correlation between porosity, stage of cavitations and fractal dimensionality. Furthermore, in porous materials, the linear, area and volumetric porosities are alike, and therefore the 3D fractal dimensionality is similar to the area one. The dimensionality of a surface is equal to two for an ideal solid (Euclidean surfaces) and equal to three for completely porous surfaces with a fractal character. Areas with *Z_i_* values above a threshold *Z* height are known as “islands”, while those with *Z*_i’_s below the threshold height value are named as “lakes”. AFM “island-lake structure” of non-irradiated and VUV irradiated 2 μm × 2 μm areas are shown in Figure 3.

**Figure 4:**
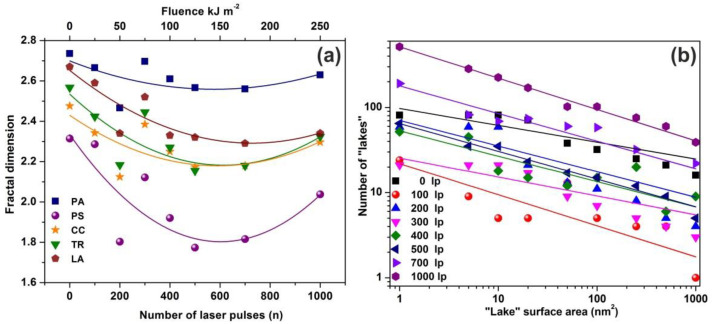
Fractal dimensionality, “lake” surface area and lake number vs. laser fluence. (**a**) Surface -fractal dimensionality calculated with four different fractal analytical methodologies. Colors, symbols and lines to assist the eye: blue squares for partitioning (PA), purple spheres for power spectrum (PS), dark yellow stars for cube counting (CC), green triangles for triangulation (TR), and brown pentagons for atomic force microscopy (AFM) “lake” pattern (LA). The five methods show a similar fractality trend vs. laser fluence; (**b**) “lake” surface area vs. the number of lakes at different number of laser pulses (laser fluence).

The mean *Z_i_* heights of non-irradiated and the irradiated regions (10^3^ laser pulses) were set at 0.75 and 1.94 nm respectively, and the irradiated areas show a diverging surface topology, in agreement with previous results [13,16,56,58]. Following a standard procedure, two parameters, the fractal dimensionality *D_f_* (which is a dimensionless number) and the “periphery to the area ratio” (PAR) are used to describe a set of “islands” or “lakes”. Both parameters are linked to the surface roughness, cavitations and topological entropy [27,74]. PAR is the ratio of logarithms of the perimeter *Π* to the area *A*, where $\Pi =\alpha \left(1+D_{f}\right)A^{\left(1-D_{f}\right)/2}$. For assessing the state of cavitations, the fractal dimensionality is calculated by the partitioning, the cube counting, the triangulation, and the power spectrum algorithms [58]. Results are compared with those derived directly from the AFM “lake” pattern software, Figure 4a.

**Figure 5:**
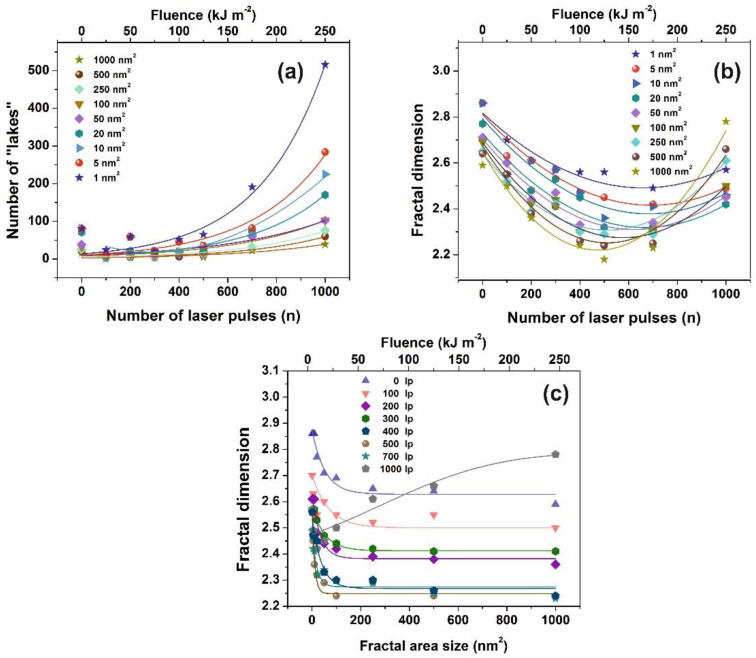
Fractal parameters of polyacrylamide (PAM) at different laser fluence: (**a**) Number of ‘‘lakes’’ for different fractal size vs. the number of laser pulses *(n)*; (**b**) fractal dimensionality vs. the number of laser pulses *(n)* for different fractal size. The concentration of small size nanocavities increases at higher laser fluence; (**c**) fractal dimension vs. fractal size at a different number of laser pulses (lp).

AFM images of 2 μm × 2 μm laser-irradiated areas were digitized to a 512 px × 512 px matrix, and then they processed with four different fractal algorithms. It is unveiled that fractal dimensionality, and thus cavitations, are functions of the laser photon fluence. All algorithms exhibit a similar trend of fractal dimensionality with the number of laser pulses (laser fluence), although the fractal dimensionality derived with the power spectra methodology seems slightly different, as expected [13,25]. The fractal dimensionality initially dips, attaining its minimum value around 500 laser pulses and then rises again with a small gradient up to 10^3^ laser pulses, Figure 4a. For a constant “lake” surface area the number of “lakes”, and thus the number of cavities, is a function of the laser pulses (laser fluence), Figure 4b. The number of “lakes” within a given surface area vs. the number of laser pulses is shown in Figure 5a. The number of “lake” areas rises almost exponentially with the number of laser pulses and small area “lakes” prevail over larger ones The fractal dimensionality vs. laser fluence has a non-monotonous complex structure. Small size features (1–10^2^ nm^2^) are associated with nanocavity-like structures, Figure 5b. It is also confirmed that below 10^3^ laser pulses small size features contribute to a high cavitation state because small size features have a higher dimensionality than large size structures, Figure 5c. On the contrary, large size features are prominent at 10^3^ laser pulses, indicating the complexity of the associated processes. In addition, for the same number of laser pulses, small size cavitation prevails over larger ones, Figure 5a. The experimental results indicate that water confinement is rather associated with small cavitations, in agreement with WLRS measurements (vide infra).

### 3.3. AFM-NI

**Figure 6:**
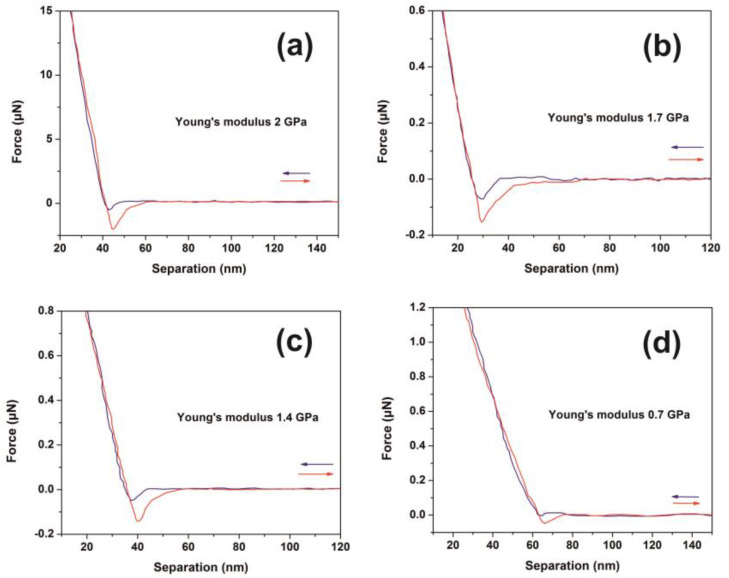
Typical force-distance (F-D) curves of polyacrylamide (PAM) thin layer surfaces (426 nm) irradiated with a different number of laser pulses (lp) (250 J m^−2^ per laser pulse): (**a**) F-D curves of the non-irradiated layer; (**b**) F-D curves with 200 lp; (**c**) F-D curves with 300 lp; (**d**) F-D curves with 400 lp.

The mechanical response of 426 nm-thick PAM polymers was evaluated with nanoscale resolution via the F-D curves at different laser fluence, Figure 6a–d. Young’s modulus and adhesion forces were also evaluated. Major non-monotonic modifications were recorded indicating substantial conformational changes of the surface energy of the PAM layers, Figure 6a. A diverging Young’s modulus is attributed to accelerated ageing because of molecular bond breaks, accompanied by the formation of new carbon and carbonyl bonds [75,76,77]. A nonlinear alteration of the elastic modulus of PAM gel formulations during ten days ageing was also reported, revealing substantial changes of PAM’s mechanical properties during irradiation [78].

The approach and retract curves follow different paths in all irradiating conditions, describing thus a system evolving out of equilibrium. The elastic modulus of the dry state hydrogels is significantly reduced after immersion to water, e.g., from 18 GPa to 3.3 MPa [79]. The Young’s modulus of the non-irradiated PAM hydrogels depends on the hydration conditions, e.g., it decreases from 295 MPa in the dried state to 266 kPa in the fully hydrated state [78,80]. A Young’s modulus of 2.84 GPa of uncured PAM hydrogel was recently attributed to the presence of pre-polymerized PAM oligomers [81]. Moreover, enhancement of Young’s modulus to 4.84 GPa was predicted via an extension of the 3D polymeric networks at higher cross-linking states [81].

**Figure 7:**
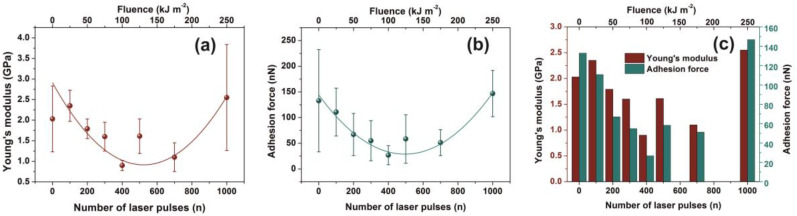
Young’s modulus and adhesion force of irradiated polyacrylamide (PAM) surfaces showing enhanced carbonization at higher laser fluence. (**a**) Young’s modulus; (**b**) adhesion force of a PAM surface irradiated at different laser fluence up to 250 J m^−*2*^; (**c**) Young’s modulus and adhesion force column charts of PAM vs. laser fluence.

In this work, the non-irradiated PAM surfaces were thermally cured after being spin-coated on a silicon wafer; therefore, their mechanical properties are expected to deviate from those in the gel state. The average Young’s modulus prior to and post-irradiation with 500 and 10^3^ laser pulses was 2.0 ± 0.8 and 1.6 ± 0.42 and 2.55 ± 1.29 GPa, respectively, Figure 7a. The significant errors of Young’s moduli at different points in the same sample are credited to various morphological heterogeneities and a progressive phase transformation to a relatively high carbonized state. Young’s moduli follow a similar trend with fractal dimensionality vs. laser fluence, Figure 4a and Figure 5b.

Additionally, the adhesive force, as it is measured during the penetrating state of AFM’s tip, follows a similar trend with Young’s modulus, Figure 7b,c. Because of diverging surface carbonization, the adhesive force drops from 130 to 26 nN between 0–400 laser pulses and then it rises again to ~ 150 nN for 10^3^ laser pulses. 

### 3.4. Water Contact Angle (CA)

**Figure 8:**
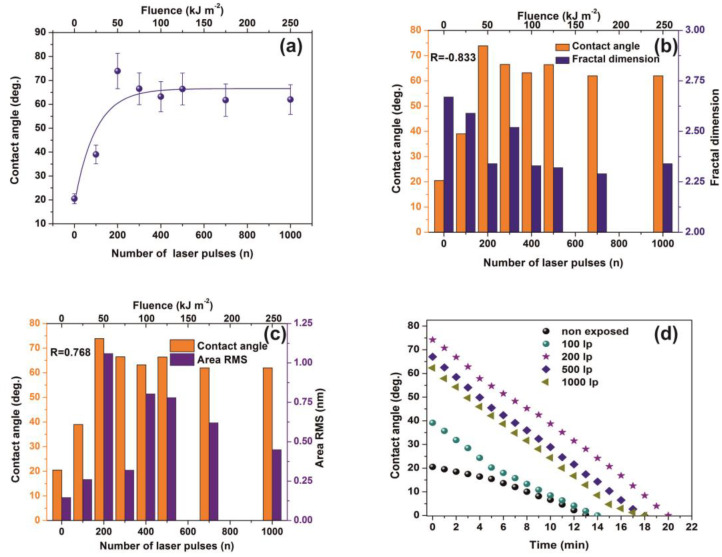
Water contact angle (CA) vs. the number of laser pulses: (**a**) Water CA vs. the number of laser pulses. (**b**) column chart diagram of CA and fractal dimensionality vs. laser fluence. The mean correlation factor is −0.833; (**c**) column chart diagram of CA and area RMS of PAM vs. laser fluence. The mean correlation factor is 0.768 pointing to a strong correlation between fractal dimensionality, CA, and area RMS over a wide range of laser fluence; (**d**) column chart diagram of water CA at different time intervals. The almost similar slopes point to a uniform surface response at different VUV photon fluence. From Figure 4a, Figure 5a,b, Figure 7a,b and Figure 8a the surface chemical modification is saturated at ~500 laser pulses, because of the low penetrating depth of the 157 nm laser photons, indicating the strong correlation between fractal dimensionality, CA, area RMS, Young’s modulus and surface modification.

Water CAs of PAM matrixes were recorded for varying photon fluence. The average CAs rise from 20° ± 2° to a saturated “plateau” at ~65°± 7° after 200 laser pulses, Figure 8a.

VUV photon processed PAM matrixes attain higher CA values, displaying thus a hydrophobic state, affirming that VUV irradiation has a primary effect on the surface wettability by altering both the material’s physicochemical properties and surface nano/micro features, Figure 8a,b. In addition, the mean correlation factors of −0.833 and 0.768 between CA, D_f_ and area RMS indicate a secure interconnection between surface morphology and *D_f_*, Figure 8b,c.

The wetting behavior was also analyzed with time, Figure 8d. The CAs of non-irradiated and irradiated with 100 laser pulses matrixes decrease consistently for 5 min. The dynamic CA of irradiated samples exhibits similar slope values, suggesting similar diffusion constants for different porosities, a fact that stresses out a picture of molecular water confinement in nanocavitations.

### 3.5. White Light Reflectance Spectroscopy (WLRS) 

WLRS uses a broad-band light source and a spectrometer. The white light emitted from the light source is guided to a reflection probe through a number of optical fibers that incident vertically onto a sample. The sample consists of a stack of transparent and semi-transparent films placed over a reflective substrate. A reflection probe collects the reflected light through a fiber, directing it to the spectrometer. The light source beam interacts with the sample and generates a reflectance signal that is constantly recorded by the spectrometer. The number and the shape of interference fringes, registered in the CCD of the spectrometer, depend on the thickness and the refractive index of the film(s). The fitting of the experimental spectrum is performed by using the Levenberg-Marquardt algorithm. 

**Figure 9:**
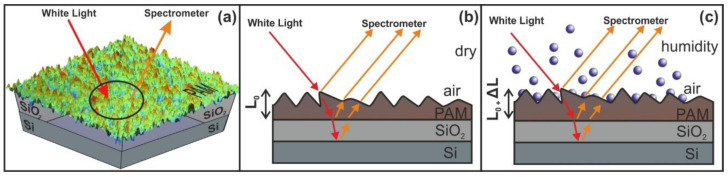
Principle of operation of white light reflectance spectroscopy (WLRS). (**a**) White light beam reflection in PAM surfaces. (**b**) experimental details and geometry of the reflected beams (**c**) surface strain response during water confinement in nanocavities. The contribution of the SiO_2_ layer at the interference pattern is negligible.

Water confinement is a source of volume strain and the relative surface deformation of the PAM polymeric matrixes caused by molecular water confinement is monitored by WLRS, Figure 9. The layer’s thickness during water confinement and the relative surface deformation of the PAM layer prior and after water confinement in the irradiated surfaces is calculated from the phase shift and the superposition of amplitudes of the reflected light beam on the PAM surfaces. The white light beam records the surface strain within a cylindrical volume ~ *V* = 4.09 × 10^−14^ m^3^, defined by the cross-sectional diameter of the white light beam of 3.5 × 10^−4^ m and the thickness of the polymeric layer of 426 nm. 

### 3.6. Random Walk Model

**Figure 10:**
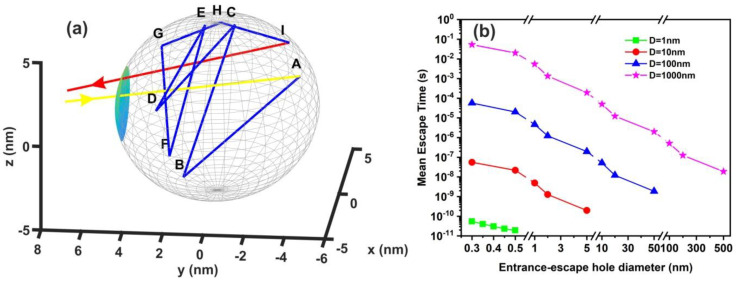
Non-interactive random walk of one water molecule in a nanocavity. (**a**) The water molecule enters the cavity (yellow arrow) and then it collides with the inside walls of the spherical cavity (10 nm) several times (A–I points and blue lines) before escaping from the entrance-escape hole (3 nm, red line); (**b**) mean escape time for 10^3^ different random walk runs in 1 nm (green), 10 nm (red), 10^2^ nm (blue), and 10^3^ nm (magenta) spherical cavities for different entrance-escape hole diameters (0.3 nm–500 nm). The y-axis represents a logarithmic time scale.

The comparison between calculations with the diffusion model [82] (water vapour diffusion coefficient in the air at normal pressure at 293 K is ~ 2.42 × 10^−5^ m^2^ s^−1^) and the current non-interactive random walk model for 10^3^ runs is shown in Table A1 and Table A2. There is a noticeable difference between the two models for small size nanocavities because the diffusion constant for small size nanocavities is undetermined. The mean escape time from random walk models with the interactive model for the different number of confined molecules, cavity and the entrance-escape hole size is given in Figure 10 and Appendix A, Table A3, Table A4, Table A5 and Table A6. 

## 4. Discussion

### 4.1. 157 nm Molecular Photodissociation of PAM Polymeric Chains 

Initially, surface and fractal analytical methods were used to typify surface cavitations crafted by 157 nm laser photons on PAM surfaces. Diverging texture morphologies of 2 μm × 2 μm PAM areas irradiated with 157 nm with a different number of laser pulses (photon fluence) are shown in Figure 2. Major conformational changes of photon processed PAM surfaces are evident through a diversity of fractal dimensionalities and surface parameters. Specifically, irradiated areas exhibit either a uniform or heterogeneous surface structural networks, according to the laser fluence (Figure 2b–e). Different size nano/microstructures including “hills and lakes” and fractal dimensionality diversity, nano aggregations (1–10^3^ nm) and cavitations are shreds of evidence of significant photochemical topological matrix alterations (Figure 2c–e). Similar structures were previously observed on PAM hydrogel surfaces by cross-link concentration variations [78].

**Figure 11:**
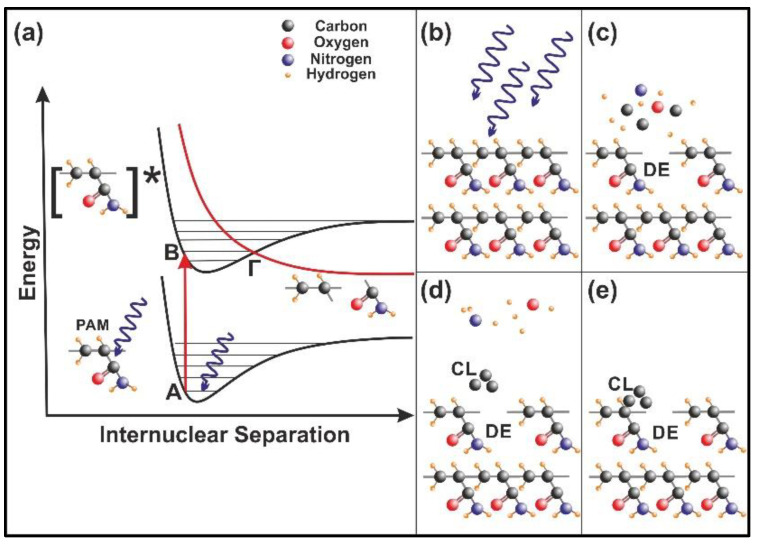
Nanocavitation by 157 nm laser photodissociation of polyacrylamide (PAM) matrixes: (**a**) Molecular photodissociation at 157 nm. Vertical arrows indicate photon transitions between two vibrational levels of the ground (A) and an excited electronic state (Β). A transition from the excited to a repulsive electronic state (red curve) through an avoided crossing via a vibration state at the point (Γ) is very fast (< 1 ps) and breaks a molecular bond in the polymeric chain; (**b**) PAM surface irradiation with 157 nm photons; (**c**) (DE): a bond break is followed by molecular decomposition and nanocavitation; (**d**) (CL): possible recombination of carbon dissociative products, and cluster formation in the gas phase; (**e**) carbon cluster deposition on the polymeric matrix and possible structure of carbon nanocavitation.

The energy of 157 nm laser photons is used to excite a molecular site in the polymeric chain from an electronic ground state (A) vibrational level to an excited electronic state (B) vibrational level, Figure 11a. The excitation is followed by a rapid internal transition to a dissociative (repulsive) state (Γ), and the parent molecule is disintegrated fast to a number of smaller size photo-fragments, Figure 11b,c. Consequently, surface irradiation with 157 nm laser photons modifies the morphology of the PAM matrix by creating defective molecular sites (DE) and micro/nano cavitations, Figure 11c. The volatile compounds, such as carbon-hydrogen monomers, ions, or larger polymer fragments, are moving away from the matrix at high velocities [37,41,42]. Carbon cluster (CL) formation (Figure 11d) also appears on the surface from re-deposited photo-dissociated products on the matrix (Figure 11e) and the photo-dissociated cycle profoundly modifies the chemical and the morphological features of the exposed polymeric surface. Because each 157 nm laser photon destroys via photo-dissociation one chemical bond of the polymeric matrix, Figure 11, it is reasonable to accept diverging cavitations and local nano-matrix volume diversities [37] in agreement with surface and fractal analysis results.

### 4.2. Trapping of Water Molecules in Nanocavities

**Figure 12:**
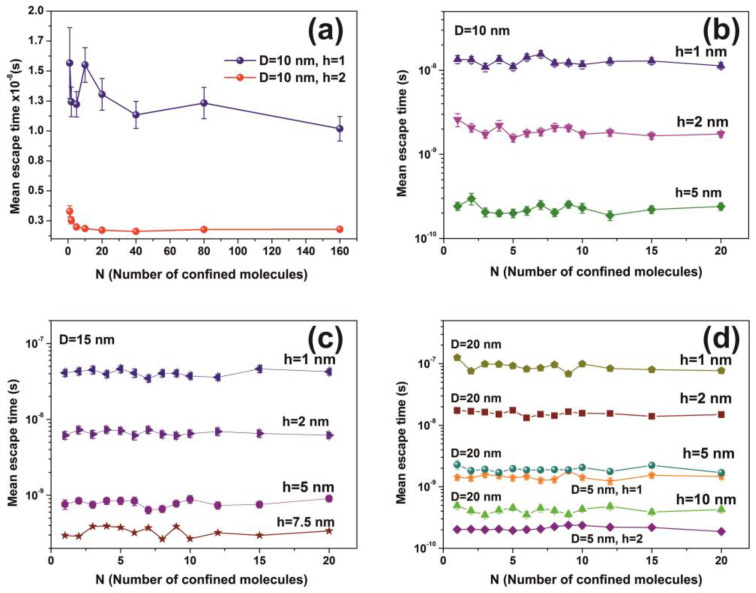
Mean escape time for a different number of non-interactive molecules calculated for 10^3^ runs. (**a**) Fluctuations of mean molecular escape time are prominent for small size cavities, while remains constant for a large number of molecules; (**b**–**d**) mean escape time for 5, 10, 15 and 20 nm spherical cavities with different entrance-escape holes and number of molecules in the cavity. The mean escape time is independent of the number of molecules and is a function only of the cavity geometry (diameter and entrance-escape hole).

Water molecular confinement is a complex issue having great importance in life sciences [8,83,84]. Because the nature of the H-bond undergoes a diverging number of structural conformations at surface boundaries, and also, inside tiny spaces, the water confinement is hindered with the long-range fluctuations of both the water networks [85] and single molecules, Figure 12a. The dynamics and the time scale of interactions in confined spaces are notably diverging with the spatial scale length and the local geometries. For example, terahertz spectroscopy of water molecules in gemstone nanocavities identify quantum water molecular tunnelling through a six-well potential caused by the interaction of the water molecule with the cavity walls [86]. The length and directionality of H-bonds are highly susceptible to the type of confining surfaces and the degree of confinement [87]. In addition, atomistic molecular dynamics simulations of dipolar fluids confined to spherical nanocavities of radii ranging from 1 to 4 nm reveal a surprisingly small Kirkwood correlation factor in water, but not so in a dipolar fluid because of ultrafast relaxation of the total dipole moment time correlation function of water [6]. The static dielectric constant of confined water exhibits a strong dependence on the size too, with a remarkably low value even at 3 nm and a slow convergence to the bulk value because of surface-induced long-range orientation correlations [6]. The trapped water experiences peculiar thermodynamic properties and under confinement unexpectedly shows high pressures (GPa) [88]. Because the mean escape time is independent of the number of molecules, inside the cavity, Figure 12, the average mechanical pressure exerted on the walls of the cavity is independent of the number of molecules. Therefore, the molecular state inside the cavity deviates from an equilibrium thermodynamic state because the escape time in “equilibrium thermodynamic” cavities should be pressure dependent. In addition, the extensive thermodynamic properties of confined molecules in tiny spaces might be disproportional to the volume of the system, and instead, they could be higher-order functions of size and shape [89,90,91,92].

It is also known that for tiny empty spaces, equal or below the atomic dimensions, stressing fields are emerging from electromagnetic vacuum fluctuations. The repulsive Casimir stress $\sigma_{c}\left(R,t\right)\;$within a conductive spherical cavity of radius R at time *t* was calculated to be $\frac{0.09\hbar c}{8\pi R^{4}\left(t\right)}$ [93]. For balancing the Casimir stress with the atmospheric pressure, a 10 nm spherical cavity has the proper size, if the equation of ideal gases is used. On the other hand, for a spherical cavity in thermal equilibrium with the matrix that bears a small hole on its surface connecting the inside with the outside volume space of the cavity, in the case of a pressure balance outside and inside the cavity, four molecules are confined in the cavity, if the equation of ideal gases is used as a first approximation. However, for an average molecular thermal energy of $E_{kin}\sim kT$ and a spherical volume *V* of 5.34 × 10^−25^ m^3^, the volume stress exerted on the walls of the cavity from the collisions of a molecule with the walls of a cavity should be of the order of $\sim \frac{kT}{V}=7.9\times 10^{4}\;\mathrm{Pa}$, a value that almost matches the atmospheric pressure outside the cavity. By increasing the number of molecules inside a small cavity, the volume stress should be increased proportionally to the number of molecules because of mechanical collisions with the cavity walls. Consequently, extremely high pressures should be developed inside small cavities, in agreement with [88]. In addition, for small size cavities, there is rather an entropic than an energy barrier that balances the flow kinetics of molecules in and out the cavity [94,95]. Previous studies indicated that in the case of elastic collisions in the cavity, the molecular dynamics depends on the number of molecules inside the cavity and is either frictionless (inertial dynamics), moderately frictional (Langevin dynamics), or strongly frictional (Brownian dynamics) [96], where the noise term should be properly taken into account. For small entrance-escape holes, the number correlation function generally decays exponentially with time. The transition rate in the frictionless limit is given by a microcanonical ensemble. As the strength of the friction is increased, the rate of collisions approaches the diffusive limit without a Kramers turnover. In this work, random-walk calculations of non-interactive and interactive molecules in the cavity for 10^3^ and 10^2^ runs, point to variable escape times of water molecules from different size nanocavities (1–10^3^ nm) and entrance-escape holes (0.3 – 5 × 10^2^ nm), Figure 10 and Appendix A, Table A1 and Table A2. For the same cavity size the mean escape time falls with large entrance-escape hole size, extended over a wide dynamic range of 10 orders of magnitude. The mean escape time for the interactive model is independent of the number of molecules inside small cavities and interestingly, the mean escape time fluctuates a great deal inside tiny cavities, Figure 12a–d and Table A3, Table A4, Table A5 and Table A6; suggesting that the system is in non-thermal equilibrium, a state that dominates the statistics and the dynamics of molecules inside small cavities. 

**Figure 13:**
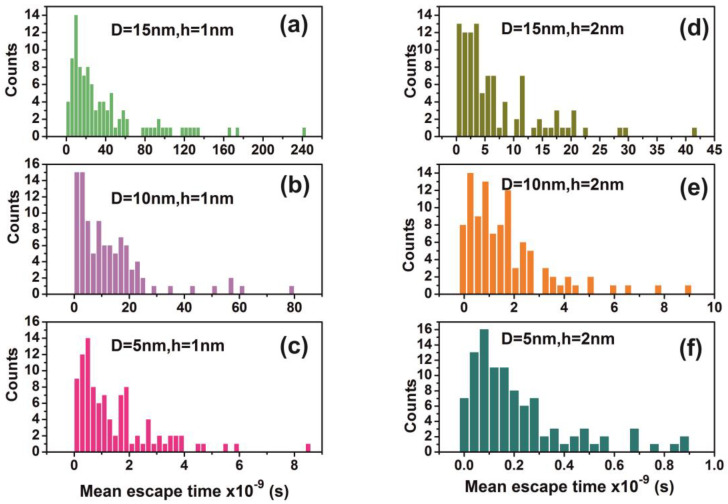
Distribution of mean escape times for 10^2^ random walk runs of a water molecule (non-interactive model) for different cavity geometries (cavity size D and entrance-escape hole size h). (**a**) D = 15 nm, h = 1 nm; (**b**) D = 10 nm, h = 1 nm; (**c**) D = 5 nm, h = 1 nm; (**d**) D = 15 nm, h = 2 nm; (**e**) D = 10 nm, h = 2 nm (**f**) D = 5 nm, h = 1 nm. Distributions are non-normal and besides that skewness and long tails indicate non-equilibrium processes inside the cavities.

**Figure 14:**
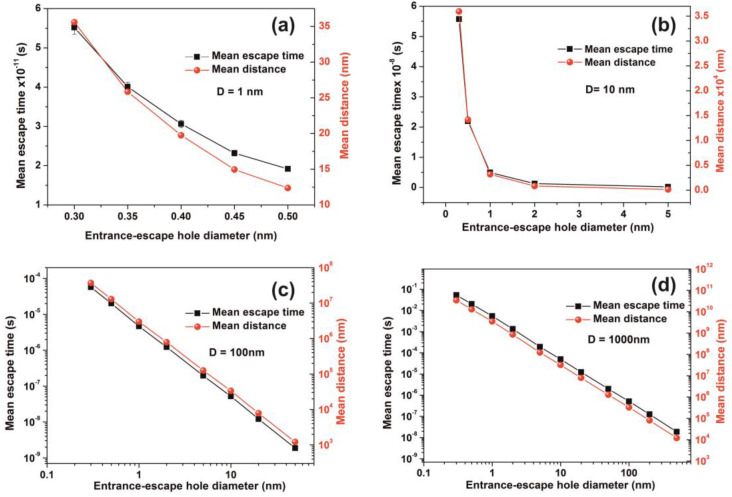
Mean escape time and mean travelling distance of a molecule within cavities of different geometries. (**a**,**b**) Gradient of the mean escape time and the mean distance that a molecule travels in the cavity before it escapes with different entrance-escape holes is diverging for very small size cavities (1, 10 nm); (**c**,**d**) mean escape time and the travelling distance gradients are constant for large size cavities (10^2^, 10^3^ nm), suggesting a non-thermal equilibrium state and large fluctuations for small size cavities.

On the contrary, for larger size cavities the volume stress is diminished and the state of molecules inside the cavities approaches the thermodynamic limit, in agreement with [86]. he gradient of the mean escape time, the mean escape time distribution and the mean distance a molecule travels inside a cavity before its escape through the entrance-escape holes is diverging for very small size cavities (1 nm) cavities, Figure 12a, Figure 13 and Figure 14a. 

**Figure 15:**
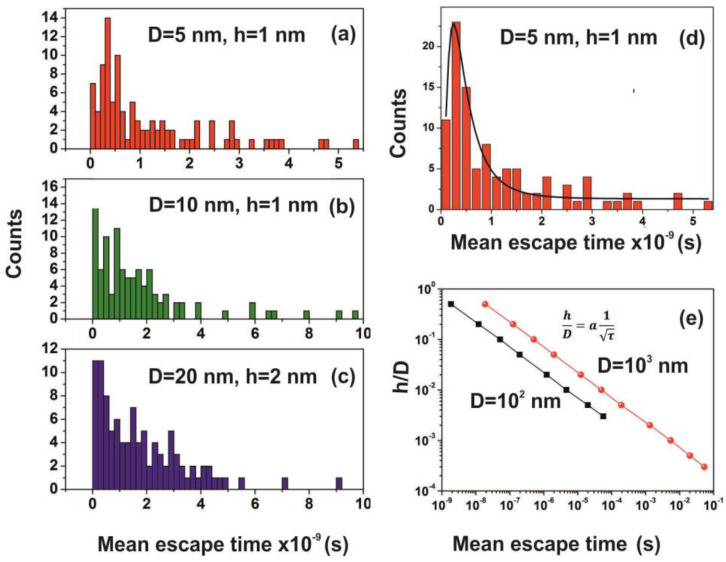
(**a**–**c**) Mean escape time distribution of 150 interactive molecules for different cavity and entrance-escape hole size for 10^2^ runs; (**d**) best-fitting of Figure 15a is for a log-normal distribution; (**e**) mean escape time vs. the cavity ratio $\frac{h}{D}$. The time differentiation of molecular movements inside and outside cavities is provided by the dependence of the ratio $\frac{h}{D}$ on the waiting time τ, which for D=10^2^ and 10^3^ nm cavities goes as a power law of the waiting time with exponent −0.5.

The mean escape time and the mean travelling distance retain a constant ratio for large size cavities (10–10^3^ nm), while the ratio deviates for small ones, suggesting again a non-equilibrium thermal state and large fluctuations inside small size cavities, Figure 14. Most interesting, the local fluctuations of mean molecular escape time are prominent for small size cavities and small number of molecules, while the mean molecular escape time remains steadier for a larger number of molecules, Figure 12a–d, in agreement with molecular dynamic results [6,85,89,90,91,92,96] and general nanothermodynamic considerations [31]. In addition, the mean escape time distribution of molecules for both the non-interactive and interactive models (1 and 150 molecules) inside different size small cavities reveals a rather non-thermal distribution and the absence of a thermal equilibrium state inside the cavities, Figure 13 and Figure 15.

The mean escape time of water molecules in the cavity is diverging from the mean collision time (~70 ns) and the thermal de Broglie time outside the cavity by many orders of magnitude, according to the geometry of the cavity. Therefore, the “molecular time” inside the cavities “flows differently” than the physical time of the events on the PAM surface. This spatial “time differentiation” across a thin layer in the PAM surface is responsible for the excess entropic potential, arising from a state of ordered arrangements between nanocavities and the water molecular ensemble of fixed molecular length near the PAM surface after 157 nm irradiation. A further sign of time differentiation of molecular movements inside and outside cavities is provided by the dependence of the ratio $\frac{h}{D}$ on the waiting time. It goes as a power law of the waiting time with exponent −0.5, Figure 15. Finally, the configured number of microstates from confinement properly counts for the experimental surface entropy deviation during the trapping of water molecules (vide infra). 

### 4.3. Stress-Strain Response in Polymeric Matrixes-A Relation between Physics and Mechanics 

During the interaction of a system with a thermal bath, the exchange of energy appears in the form of heat or work. The first law of thermodynamics states that the infinitesimal change of the heat absorbed by a system per unit volume Q is equal to the increase of the differential of its internal energy change U minus the infinitesimal change of the work done on the system *W*:

(2) δQ=dU−δW

The departure from a state of equilibrium will be governed by the second law of thermodynamics and the direction of entropy change. Any thermodynamic system is described by its extensive and intensive variables, $U,\;S,\;\sigma_{ij},\;e_{ij},\;T,$ where U is the internal energy, S is the entropy, $\sigma_{ij}\;$and $\varepsilon_{ij}\;$stand for second-rank stress and strain tensors acting on the volume element dV and T is the temperature of the system. Following Cauchy’s theory of stress, the equilibrium of elements requires the balance of forces acting on a volume element, [97]

(3) $$t_{i}^{n}=\frac{d{\vec{F}}_{i}}{dA}=\sigma_{ji}n_{j}$$

$t_{i}^{n}\;$is the *i^th^* component of a traction force$\;d\vec{F}$ along the *i-*axis, along a unit vector $\vec{n}$ perpendicular on an infinitesimal surface area $d\vec{A}$,$\;\sigma_{ij}\;$is the (i, j) component of the stress tensor, and$\;n_{j}$ is the *j_th_* component of the $\vec{n}$ vector that is perpendicular to the surface area $d\vec{A}$.

The force component$\;F_{i}$ acting on the volume element *dV* and bounded by the surface area *A* is given by the equation

(4) $$F_{i}=\int_{A}t_{i}^{n}\;dA+\int_{V}X_{i}dV\;\;\;$$

where *X_i_* are the body forces (e.g., the weight of the volume element *dV*). From Equation (4) and the Gauss theorem, the surface integral of the components of the traction forces is transformed into a volume integral

(5) $$F_{i}=\int_{V}\left(\frac{\partial t_{i}^{n}}{\partial x_{i}}+X_{i}\right)dV=\int_{V}\left(\frac{\partial \sigma_{ji}}{\partial x_{j}}+X_{i}\right)dV\;\;\;$$

For the infinitesimal theory of elasticity the strain tensor $\varepsilon_{ij}$ is reduced to a linear form

(6) $$\varepsilon_{ij}=\left(\frac{\partial u_{i}}{\partial x_{j}}+\frac{\partial u_{j}}{\partial x_{i}}\right)\;\;\;$$

where the displacement gradient of the volume element dV along one axis $\frac{\partial u_{i}}{\partial x_{j}}$ is a second-order tensor, and the derivative of $u_{i}$ is a second rank tensor

(7) $$\frac{\partial u_{i}}{\partial x_{j}}=\frac{1}{2}\left(\frac{\partial u_{i}}{\partial x_{j}}+\frac{\partial u_{j}}{\partial x_{i}}\right)+\frac{1}{2}\left(\frac{\partial u_{i}}{\partial x_{j}}-\frac{\partial u_{j}}{\partial x_{i}}\right)=\frac{1}{2}\varepsilon_{ij}-\frac{1}{2}\omega_{ij}$$

where $\omega_{ij}=\frac{\partial u_{i}}{\partial x_{j}}-\frac{\partial u_{j}}{\partial x_{i}}$ is the rotational skew-symmetric tensor.

The infinitesimal displacement $du_{i}$ along the *j* direction and for$\;\omega_{ij}=0$ is

(8) $$du_{i}=\frac{\partial u_{i}}{\partial x_{j}}dx_{j}=\frac{1}{2}\varepsilon_{ij}dx_{j}$$

Using Gauss’ theorem and Equations (4) and (8) the total mechanical work is done on the system by the traction and the body forces

(9) $$W=\int_{A}(t_{i}^{n}\;du_{i})dA+\int_{V}(X_{i}du_{i})dV=\int_{A}\sigma_{ji}n_{j}du_{i}dA+\int_{V}X_{i}du_{i}dV\;\;$$

For an isothermal and isobaric process during sorption, the infinitesimal mechanical work *δW* along the direction $n_{i}$ outwards the boundaries of a surface *A* enclosing the volume $dV=dx_{i}dA$ is equal with

(10) $$\delta W=(\frac{1}{2}\sigma_{ij}n_{j}\varepsilon_{ij}+X_{i}du_{i})dV$$

Neglecting the body forces $X_{i}\;,\;$the mechanical work is

(11) $$\delta W=\frac{1}{2}\sigma_{ij}n_{j}\varepsilon_{ij}dV$$

A superposition of the three normal stress components uniformly distributed over the volume dV is used to calculate the strain in a given direction, say the *z*-axis:

(12) $$\varepsilon_{z}=\frac{1}{E\left(n\right)}(\sigma_{x}+\sigma_{y}+\sigma_{z})$$

where E(n) is the modulus of elasticity in tension or Young’s modulus.

(13) $$\varepsilon_{x}=-\nu \frac{\sigma_{z}}{Ε\left(n\right)},\;\;\varepsilon_{y}=-\nu \frac{\sigma_{z}}{Ε\left(n\right)}$$

in which *ν* is a constant called Poisson’s ratio, equal to ~ 0.3. Using Equations (12) and (13) we obtain the strain equations along the principal axes

(14) $$\varepsilon_{x}=\frac{1}{E\left(n\right)}\left[\sigma_{x}-\nu \left(\sigma_{y}+\sigma_{z}\right)\right]$$

$$\varepsilon_{y}=\frac{1}{E\left(n\right)}\left[\sigma_{y}-\nu \left(\sigma_{z}+\sigma_{x}\right)\right]$$

$$\varepsilon_{z}=\frac{1}{E\left(n\right)}\left[\sigma_{z}-\nu \left(\sigma_{x}+\sigma_{y}\right)\right]$$

For relatively thick and isotropic layers and for small linear and homogeneous elastic deformations along the axes, the normal stresses are equal and Equation (14) reads as

(15) $$\varepsilon_{z}=\frac{1-2\nu }{E\left(n\right)}\sigma_{z}$$

Because a contraction of a volume element in the *z*-direction in an elastic medium is accompanied by lateral extensions

(16) $$\;\varepsilon_{z}=-\varepsilon_{x}=-\varepsilon_{y}$$

and using Equation (16) in Equation (11) the mechanical work along the principal axes is

(17) $$\delta W=\frac{1}{2}\sigma_{z}\varepsilon_{z}dV$$

From the first and second law of thermodynamics, the mechanical work W done on a system is

(18) $$W=T\;S-U+\mu_{i}N_{i\;}+\Psi A$$

and the infinitesimal mechanical work per unit volume before and after sorption is

(19) $$\frac{Ε\left(n\right)}{2\left(1-2\nu \right)}\varepsilon_{z}^{2}dV=-\delta U+T\delta S+\mu_{i}\delta N_{i}+\delta \left(\Psi A\right)$$

where δU, δS, and$\;\delta N_{i}\;$stand for the variations of the internal energy, entropy and the number of bind water molecules on active polymeric sites prior and after sorption,$\;e_{z\;}\;$is the strain of the volume element along the *z-*axis from the confinement of water molecules, $\mu_{i}$ is the chemical potential of $\delta N_{i\;}$absorbed particle on the polymeric matrix. The term$\;\delta \left(\Psi \left(n\right)\right)=\delta \left[\gamma \left(n\right)+Ε{\left(n\right)}_{s}\iint^{\;}n_{k}dA_{k}\right]$ is the algebraic sum of the surface energy δ(γ(n)) plus the elastic energy strain $Ε{\left(n\right)}_{s}$ of the nanocavities per unit area, from surface irradiation with some *n* laser pulses at 157 nm. The last term is zero under isothermal and isobaric sorption, δ(Ψ(n))=0, [97]. The term $\delta N_{i}$ is relatively negligible because of a small number of absorbed water molecules. Finally, the strain of a volume dV along the *z*-axis before and after water confinement is given by the equation

(20) $$\varepsilon_{z}={\left(\frac{2\left(1-2\nu \right)\left(T\delta S-dU\right)}{Ε\left(n\right)dV}\right)}^{1/2}$$

### 4.4. Internal Energy Variation during Molecular Water Confinement 

Besides confinement, molecular water molecules are attached to polymeric sites via electric dipole interactions. The internal energy variation is the outcome of the photon-escalating number of active dipole binding sites spawn via VUV matrix irradiation, Figure 11.

For surface irradiation with *n* laser pulses, the internal energy variation $\delta U_{b}\;$is given by the relation [13,98]

(21) $$\delta U_{b}=-\lambda lN_{b}\left(n\right)<\Phi >=-\lambda lN_{b}\left(n\right)\frac{5d_{xy}^{4}}{64\pi^{4}\varepsilon_{0}^{2}\varepsilon_{1}^{2}k_{B}Tr^{6}}$$

$N_{b}\left(n\right)$ is the number of water molecules attached on the active sites, *λ* is the probability that a water molecule will overcome an energy threshold barrier and bind in a photon-activated dipole binding site and *l* is the average number of adsorbed water molecules on each photon-activated dipole binding site. $d_{xy}$= er is the *x, y* component of the electric dipole moment between a water molecule and a photo-activated dipole binding site. $e=1.6\times 10^{-19}\;C$ is the electron charge and r~0.1 nm is a mean separating distance between a water molecule and a photon-activated dipole binding site, $\varepsilon_{0}$ is the vacuum permittivity equal to 8.85 × 10^−12^ Fm^−1^, $\varepsilon_{1}\;\sim 80$ is the relative electric permittivity of the polymer-water system, $k_{B}=1.38\times 10^{-23}\;{J\;K}^{-1}$ is Boltzmann’s constant and T = 300 K is the absolute temperature. Because the energy of each laser pulse at 157 nm is 28 mJ, the number of photons carried in one laser pulse is $n=2.26\times 10^{16}$ photons/laser pulse, and this number equals to the number of photon-activated dipole binding sites. Each VUV photon at 157 nm dissociates one molecular bond and creates one active site on the polymeric matrix, Figure 11. For a 1.12 × 10^−4^ m^2^ cross-sectional area of the 157 nm laser beam and 426 nm layer thickness, it is found that 4.73 × 10^26^ photon-activated dipole binding sites are generated within 1 m^3^ per laser pulse. For a cross-section area of the WLRS beam of 4.90 × 10^−8^ m^2^ and 426 nm matrix thickness, the volume dV of the polymeric matrix occupied by the white light beam is 4.09 × 10^−14^ m^3^ and thus the total number of active binding sites per laser pulse within the volume occupied by the white beam is *N_b_* = 2.31 × 10^13^. From Equation (21) $<\Phi >\approx 1.51\;x\;10^{-23}\;J$ for λl=0.05 (vide infra) and finally $\delta U_{d}=1.43\times 10^{-11}\;J.$

### 4.5. Entropic Energy Variation during Molecular Confinement

**Figure 16:**
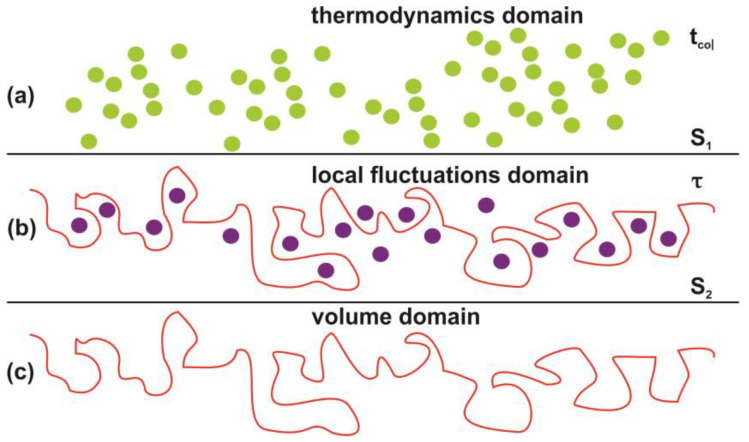
Schematic layout of the interphase between the photon processed polyacrylamide PAM surface and the water vapor domain. (**a**) Thermal equilibrium domain. Reference time and space scales are determined by the mean collision time *t_col_* between the water and air molecules and the entropy of the ideal gases and the mean collision distance. The entropy *S_1_* is given by the Sackur–Tetrode equation for the ideal gases [99]; (**b**) local fluctuations domain. Nanocavitations on the surface with confined molecules. The time scale is determined by the mean escape time *τ* of water molecules. The entropy *S_2_* in this domain is determined by the number of microstates *Ω(Ν_b_(n), N_c_(n), Ε_α_)*, which specify a state of ordered arrangements between nanocavities in one hand and molecular water ensembles of fixed molecular length near the surface on the other; (**c**) volume matrix domain.

Photon-induced nanocavitations are also responsible for surface entropic variation at the boundary between inside and outside nanocavity areas. The entropic variation at the interphase has its origin from time differentiation between the inside and outside areas of nanocavities. Actually, the mean collision time (~70 ns) of water molecules outside the nanocavities within the matrix or near the surface is specified by the laws of ideal gases. On the contrary, the mean escape time of water molecules inside the nanocavities is specified by the hole geometry and the interplay between entrance-escape hole size with cavity diameter. The waiting times follow an inverse power law behavior because thermal equilibrium does not apply in tiny spaces, Figure 12, Figure 13, Figure 14, Figure 15 and Figure 16 and Table A1 and Table A2. In addition, VUV laser irradiation locally ablates the polymeric material, crafting photon-guided “hill-lake” morphologies. The total number of lakes (cavities) vs. the surface area follows a power-law behavior. In this dependency, the number of laser pulses is present through a pre-factor term, Figure 4b. A schematic layout of this modified interphase between photon processed PAM surface and water vapor domain is illustrated in Figure 16. 

Random movements in such complex landscapes could be modeled in the frame of continuous time random walk [100,101] by also taking into account the fractal properties of the modified polymeric material [102]. We leave this challenging task for future work where both analytical and extensive numerical calculations combined with experimental results will be presented. Because different water molecules enter and escape the nanocavitations, the number of different microstates *Ω(Ν_b_(n), N_c_(n), Ε_α_)* per unit time is specified by the frequency of water molecules confined in the nanocavities. The rate of visits is regulated by the mean escape time of water molecules. *n* and $N_{c}\left(n\right)\;$is the number of laser pulses and nanocavities, respectively, *N_a_* is the number of water molecules outside the nanocavities with energy *Ε_α_* and *N_c_(n)* is the number of nanocavities. The number of microstates is equal to the number of indistinguishable permutations $\left\{N_{a}\left(n\right),\;N_{b\;}\left(n\right)+N_{c}\left(n\right)\right\}\;$between the number of water molecules *N_a_* and the number of nanocavities $N_{c}\left(n\right)$ and the photon-induced dipole binding sites $N_{b}\left(n\right)$

(22) $$\Omega \left({Ν}_{b}\left(n\right),\;N_{c}\left(n\right),\;{Ε}_{\alpha }\right)=\left\{N_{a}\left(n\right),\;N_{b\;}\left(n\right)+N_{c}\left(n\right)\right\}\;=\frac{N_{a}!}{(N_{b\;}\left(n\right)+N_{c}\left(n\right))!(N_{a}-(N_{b\;}\left(n\right)+N_{c}\left(n\right))!}\;\mathrm{for}\;\;\;N_{b\;}\left(n\right)+N_{c}\left(n\right)<N_{a}$$

(23) $$\Omega \left({Ν}_{b}\left(n\right),\;N_{c}\left(n\right),\;{Ε}_{\alpha }\right)=\left\{N_{b\;}\left(n\right)+N_{c}\left(n\right),N_{a}\left(n\right)\right\}\;=\frac{(N_{b\;}\left(n\right)+N_{c}\left(n\right))!}{N_{a}!((N_{b\;}\left(n\right)+N_{c}\left(n\right))-N_{a})!\;}\;\;\;\mathrm{for}\;\;N_{b\;}\left(n\right)+N_{c}\left(n\right)>N_{a\;\;\;\;}$$

To arrive in Equations (22) and (23) it is considered that only one water molecule per unit time is either trapped to a specific nanocavity or attached a photon-induced polar binding site. An escalating number of nanocavities is building up in the matrix after each laser pulse, and the ratio of the sum of the number of dipole binding sites and nanocavities to the number of water molecules near the surface is a function of the number of laser pulses

(24) $$x\left(n\right)=\frac{N_{b\;}\left(n\right)+N_{c}\left(n\right)}{N_{a}}$$

From Equations (22)–(24), the entropy from the confinement and the attachment of water molecules in nanocavities and photon-induced polar adhesion binding sites is [13,26]

(25) $$\delta S=k_{B}ln\Omega \left({Ν}_{b}\left(n\right),\;N_{c}\left(n\right),\;{Ε}_{\alpha }\right)=k_{B}ln\left\{N_{a}\left(n\right),\;N_{b\;}\left(n\right)+N_{c}\left(n\right)\right\},\;\;x\left(n\right)<1$$

(26) $$\delta S=k_{B}ln\Omega \left({Ν}_{b}\left(n\right),\;N_{c}\left(n\right),\;{Ε}_{\alpha }\right)=k_{B}ln\left\{N_{b\;}\left(n\right)+N_{c}\left(n\right),N_{a}\left(n\right)\right\},\;\;x\left(n\right)>1$$

Using Equation (22), Equation (25) read as

(27) $$\delta S=k_{B}\{\;ln(N_{a}!)-ln\left((N_{b\;}\left(n\right)+N_{c}\left(n\right)\right))!-ln(N_{a}-(N_{b\;}\left(n\right)+N_{c}\left(n\right))!$$

By using Stirling’s formula

(28) lnN!=NlnN−N

Equation (27) transforms to

(29) $$\delta S=k_{B}\{N_{a}ln(N_{a}\text{)}-N_{a}-(N_{b\;}\left(n\right)+\;N_{c}\left(n\right))\;\text{ln}\left(N_{b\;}\left(n\right)+\;N_{c}\left(n\right)\right)+\left(N_{b\;}\left(n\right)+\;N_{c}\left(n\right)\right)\;-(N_{a}-(N_{b\;}\left(n\right)+\;N_{c}\left(n\right)\text{ln}(N_{a}-(N_{b\;}\left(n\right)+\;N_{c}\left(n\right)+(N_{a}-(N_{b\;}\left(n\right)\;+\;N_{c}\left(n\right)))\}\;\;=k_{B}\{N_{a}ln(N_{a}\text{)}-(N_{b\;}\left(n\right)+\;N_{c}\left(n\right))\;ln\left(N_{b\;}\left(n\right)+\;N_{c}\left(n\right)\right)\;-(N_{a}-(N_{b\;}\left(n\right)+\;N_{c}\left(n\right))\ln(N_{a}-(N_{b\;}\left(n\right)+\;N_{c}\left(n\right))\}$$

Using Equation (24), Equation (29) becomes

(30) $$\delta S=k_{B}\left(N_{b}\left(n\right)+N_{c}\left(n\right)\right)\left\{\ln\left(\frac{1-x\left(n\right)}{x\left(n\right)}\right)-\frac{1}{x\left(n\right)}\ln\left(1-x\left(n\right)\right)\right\},\;\;x\left(n\right)<1\;\;$$

Similarly from Equations (23) and (24)

(31) $$\delta S=k_{B}\left(N_{b}\left(n\right)+N_{c}\left(n\right)\right)\left\{\ln\left(\frac{x\left(n\right)}{x\left(n\right)-1}\right)+\frac{1}{x\left(n\right)}\ln\left(x\left(n\right)-1\right)\right\},\;\;\;\;\;\;\;x\left(n\right)>1\;\;$$

In the case of a constant attachment of water molecules in the photon-induced binding sites, Equations (30) and (31) are modified accordingly

(32) $$\delta S=k_{B}N_{c}\left(n\right)\left\{\ln\left(\frac{1-y\left(n\right)}{y\left(n\right)}\right)-\frac{1}{y\left(n\right)}\ln\left(1-y\left(n\right)\right)\right\},\;\;y\left(n\right)<1$$

(33) $$\delta S=k_{B}N_{b}\left(n\right)\beta \left(n\right)\left\{\ln\left(\frac{y\left(n\right)}{y\left(n\right)-1}\right)+\frac{1}{y\left(n\right)}\ln\left(y\left(n\right)-1\right)\right\},\;\;\;\;\;\;\;y\left(n\right)>1\;\;$$

where

(34) $$y\left(n\right)=\frac{N_{c}\left(n\right)}{N_{a}},\;\;N_{c}\left(n\right)=\beta \left(n\right)N_{b}\left(n\right)$$

In the case where some nanocavities are not visited by the water molecules, then y(n)>1 . This condition is fulfilled under the current experimental configuration, Figure 14. For β(n)~0.2, *N_b_* = 2.31 × 10^13^, y(n)=2, the entropic energy at 300 K is $k_{B}T\delta S=1.31\times 10^{-8}\;J$, which is almost three orders of magnitude larger than $\delta U_{d}.$ Equations (32), (33) properly reflect the extensive variable character of the entropy as it should be.

### 4.6. Surface Strain from the Confinement of Water Molecules 

Using Equations (20) and (32)–(34) the surface strain following 157 nm laser irradiation takes the form

(35) $$\varepsilon_{z}={\left(\frac{N_{b}\left(n\right)}{E\left(n\right)dV}\right)}^{\frac{1}{2}}[-\lambda l<\Phi >+k_{B}T\beta \left(n\right)[\ln\left(\frac{1-y\left(n\right)}{y\left(n\right)}\right)-\frac{1}{y\left(n\right)}\ln\left(1-y\left(n\right)\right)]{]}^{\frac{1}{2}}\;\;\;y\left(n\right)<1$$

(36) $$\varepsilon_{z}={\left(\frac{N_{b}\left(n\right)}{E\left(n\right)dV}\right)}^{\frac{1}{2}}[-\lambda l<\Phi >+k_{B}T\beta \left(n\right)[ln\frac{y\left(n\right)}{y\left(n\right)-1}+\frac{1}{y\left(n\right)}\ln\left(y\left(n\right)-1\right)]{]}^{\frac{1}{2}}\;\;\;\;\;\;y\left(n\right)>1$$

**Figure 17:**
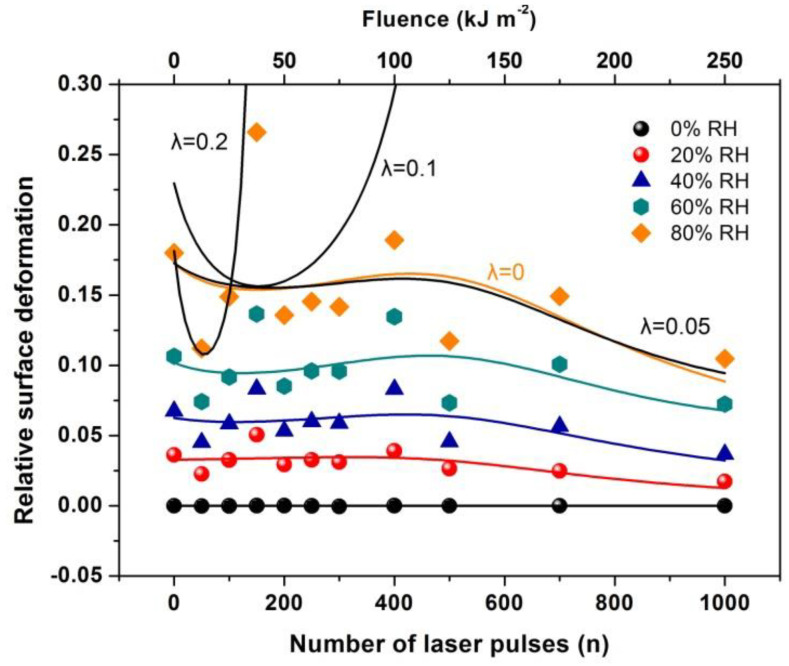
Relative surface deformation (strain) of the 426 nm polyacrylamide (PAM) layers measured with white light reflectance spectroscopy (WLRS) at different 157 nm irradiating conditions of the PAM matrix and relative humidity (RH). The solid lines at different RH represent the best fit of Equation (36) to the experimental data. The black lines at 80 % RH are the fittings for different λl values of 0, 0.05, 0.1 and 0.2. The best fit (orange line) respectively, is for 0≤λl<0.05  suggesting a small contribution to the relative surface deformation from electric dipole attachment of water molecules to active binding sites in the PAM matrix and a substantial contribution from the confinement of water molecules in nanocavities, Equation (36).

Equations (35) and (36) shape the main result. The equations relate the surface strain $\varepsilon_{z}$ and Young’s modulus E(n) with the number of nanocavities, the photon-induced dipole binding sites in the matrix, and the water vapor molecules near nanocavities. For the current experimental configuration y(n)>1. From Equation (36), the strain at 400 laser pulses is ~ 0.1 in agreement with the experimental results of Figure 17. 

By fitting Equation (36) to the experimental data of Figure 17, the functional dependence of *y(n)* on the number of photons *n* is determined at different relative humidity (RH) values. Because *y(n)* is proportional to the number of dipole binding sites and the number of nanocavities *N_c_ (n)*, *y(n)* is a measure of the surface carbonization. By using a linear functional for both *y(n)* and *E(n),* the best fit of Equation (36) to the experimental data of Figure 17 for relative humidity 80% is for β(n)=0.2 and 0≤λl<0.05. The above fitting values suggest a small and large contribution from the electric dipole interactions and the entropic variation in surface strain, respectively. From Equation (36), the surface strain is proportional to the square root of the number of nanocavities and the concentration of the water molecules (RH) and inversely proportional to the square root of Young’s modulus of the surface, in agreement with the experimental results of Figure 17. Finally, the entropic jump, probed by WLRS, trails the confinement of water molecules in nanocavities, while the deep physical root of surface entropy variation originates from the different “time flow and scales” and the validity and invalidity of thermal equilibrium outside and inside the nanocavities, respectively, Figure 15, Figure 16, Figure 17.

The experimental approach permits to monitor water confinement on surfaces, including biological ones. 

## 5. Conclusions

Water molecules confined inside laser photon crafted nanocavities on PAM polymeric matrixes are in a state of non-thermal equilibrium. The mean escape time of water molecules from the nanocavities diverges from the mean collision time of water molecules outside the nanocavities (ideal gas state). The time differentiation inside and outside the nanocavities reveals an additional state of ordered arrangements between nanocavities and molecular water ensembles of fixed molecular length near the surface. The configured number of microstates correctly counts for the experimental surface entropy deviation during molecular water confinement.

## Appendix A

**Table A1:** Mean escape time (τ) of a given molecule, errors and total traveling distance of the confined molecule in a spherical cavity (cavity diameter D of 1 nm and 10 nm) for different entrance–escape hole diameters (h) calculated by the diffusion and the non-interactive random walk models.

h (nm)	Cavity Diameter D = 1 nm					Cavity Diameter D = 10 nm				
	Diffusion	Random Walk				Diffusion	Random Walk			
	τ (s)	τ (s)	Error (s)	Distance (nm)	Error (nm)	τ(s)	τ (s)	Error (s)	Distance (nm)	Error (nm)
0.3	3.98 × 10^−14^	5.51 × 10^−11^	1.7 × 10^−12^	35.5	1.1	3.98 × 10^−11^	5.57 × 10^−8^	1.7 × 10^−9^	35,931	1133
0.5	2.39 × 10^−14^	1.91 × 10^−11^	5 × 10^−13^	12.4	0.3	2.39 × 10^-11^	2.19 × 10^−8^	7 × 10^−10^	14,151	465
1						1.19 × 10^-11^	4.93 × 10^−9^	1.6 × 10^−10^	3182	107
2						5.98 × 10^-12^	1.28 × 10^−9^	4 × 10^−11^	822	26
5						2.39 × 10^−12^	1.90 × 10^−10^	5 × 10^−12^	128	4

**Table A2:** Mean escape time (τ) of a given molecule, errors and total traveling distance of the confined molecule in a spherical cavity (cavity diameter 10^2^ nm and 10^3^ nm) for different entrance–escape hole diameters (h) calculated by the diffusion and the non-interactive random walk models.

h (nm)	Cavity Diameter D = 10^2^ nm					Cavity Diameter D = 10^3^ nm				
	Diffusion	Random Walk				Diffusion	Random Walk			
	τ (s)	τ (s)	Error (s)	Distance (nm)	Error (nm)	τ (s)	τ (s)	Error (s)	Distance (nm)	Error (nm)
0.3	3.98 × 10^−8^	5.71 × 10^−5^	1.8 × 10^−6^	3.68 × 10^7^	1.2 × 10^6^	3.98 × 10^−5^	5.3 × 10^−2^	1.7 × 10^−3^	3.42 × 10^10^	1.1 × 10^9^
0.5	2.39 × 10^−8^	2.00 × 10^−5^	6 × 10^−7^	1.29 × 10^7^	4.1 × 10^5^	2.39 × 10^−5^	2.01 × 10^−2^	6.3 × 10^−4^	1.3 × 10^10^	4.1 × 10^8^
1	1.19 × 10^−8^	4.63 × 10^−6^	1.4 × 10^−7^	2.99 × 10^6^	9 × 10^4^	1.19 × 10^−5^	5.43 × 10^−3^	1.6 × 10^−4^	3.5 × 10^9^	1.1 × 10^8^
2	5.98 × 10^−9^	1.22 × 10^−6^	4 × 10^−8^	7.89 × 10^5^	2.5 × 10^4^	5.98 × 10^−6^	1.33 × 10^−3^	4.1 × 10^−5^	8.56 × 10^8^	2.7 × 10^7^
5	2.39 × 10^−9^	1.95 × 10^−7^	6 × 10^−9^	1.26 × 10^5^	4 × 10^3^	2.39 × 10^−6^	1.92 × 10^−4^	6.7 × 10^−6^	1.24 × 10^8^	4.3 × 10^6^
10	1.19 × 10^−9^	5.18 × 10^−8^	1.6 × 10^−9^	3.34 × 10^4^	1060	1.19 × 10^−6^	4.99 × 10^−5^	1.6 × 10^−6^	3.22 × 10^7^	1.1 × 10^6^
20	5.97 × 10^−10^	1.21 × 10^−8^	3 × 10^−10^	7821	244	5.97 × 10^−7^	1.23 × 10^−5^	4.1 × 10^−7^	7.93 × 10^7^	2.6 × 10^5^
50	2.39 × 10^−10^	1.87 × 10^−9^	5 × 10^−11^	1204	35	2.39 × 10^−7^	1.99 × 10^−6^	6.4 × 10^−8^	1.28 × 10^6^	4.1 × 10^4^
100						1.19 × 10^−7^	5.07 × 10^−7^	1.5 × 10^−8^	3.27 × 10^5^	9.8 × 10^3^
200						5.97 × 10^−8^	1.25 × 10^−7^	3.7 × 10^−9^	8.05 × 10^4^	2.4 × 10^3^
500						2.39 × 10^−8^	1.88 × 10^−8^	5.6 × 10^−10^	1.21 × 10^4^	3.6 × 10^2^

**Table A3:** Mean escape time (τ) and associated errors of different number molecules confined in a 5-nm spherical cavity for different entrance–escape hole diameters (h) calculated by the interactive random walk models for 10^2^ runs.

Cavity Diameter = 5 nm				
N (Number of Molecules)	h = 1 nm		h = 2 nm	
	τ (s)	Error (s)	τ (s)	Error (s)
1	1.41 × 10^−9^	1.4 × 10^−10^	2.02 × 10^−10^	2.0 × 10^−11^
2	1.38 × 10^−9^	1.4 × 10^−10^	2.04 × 10^−10^	2.2 × 10^−11^
3	1.57 × 10^−9^	1.6 × 10^−10^	2.00 × 10^−10^	2.1 × 10^−11^
4	1.50 × 10^−9^	1.4 × 10^−10^	2.05 × 10^−10^	1.9 × 10^−11^
5	1.39 × 10^−9^	1.4 × 10^−10^	1.95 × 10^−10^	1.8 × 10^−11^
6	1.46 × 10^−9^	1.3 × 10^−10^	2.01 × 10^−10^	2.2 × 10^−11^
7	1.27 × 10^−9^	1.2 × 10^−10^	2.06 × 10^−10^	2.1 × 10^−11^
8	1.30 × 10^−9^	1.4 × 10^−10^	2.25 × 10^−10^	2.4 × 10^−11^
9	1.82 × 10^−9^	1.8 × 10^−10^	2.37 × 10^−10^	2.1 × 10^−11^
10	1.41 × 10^−9^	1.4 × 10^−10^	2.35 × 10^−10^	2.4 × 10^−11^
12	1.23 × 10^−9^	1.5 × 10^−10^	2.20 × 10^−10^	2.1 × 10^−11^
15	1.53 × 10^−9^	1.5 × 10^−10^	2.18 × 10^−10^	2.0 × 10^−11^
20	1.47 × 10^−9^	1.3 × 10^−10^	1.87 × 10^−10^	1.9 × 10^−11^

**Table A4:** Mean escape time (τ) and associated errors of different number molecules confined in a 10-nm spherical cavity for different entrance–escape hole diameters (h) calculated by the interactive random walk models for 10^2^ runs.

Cavity Diameter = 10 nm						
N (Number of Molecules)	h = 1 nm		h = 2 nm		h = 5 nm	
	τ (s)	Error (s)	τ (s)	Error (s)	τ (s)	Error (s)
1	1.35 × 10^−8^	1.5 × 10^−9^	2.59 × 10^−9^	4.6 × 10^−10^	2.42 × 10^−10^	2.7 × 10^−11^
2	1.33 × 10^−8^	1.3 × 10^−9^	2.07 × 10^−9^	2.3 × 10^−10^	2.97 × 10^−10^	4.7 × 10^−11^
3	1.09 × 10^−8^	1.3 × 10^−9^	1.74 × 10^−9^	1.7 × 10^−10^	2.06 × 10^−10^	2.5 × 10^−11^
4	1.35 × 10^−8^	1.5 × 10^−9^	2.21 × 10^−9^	3.2 × 10^−10^	2.00 × 10^−10^	1.8 × 10^−11^
5	1.10 × 10^−8^	1.1 × 10^−9^	1.57 × 10^−9^	1.7 × 10^−10^	2.00 × 10^−10^	2.4 × 10^−11^
6	1.43 × 10^−8^	1.4 × 10^−9^	1.78 × 10^−9^	1.7 × 10^−10^	2.14 × 10^−10^	2.5 × 10^−11^
7	1.55 × 10^−8^	1.6 × 10^−9^	1.85 × 10^−9^	1.9 × 10^−10^	2.51 × 10^−10^	3.1 × 10^−11^
8	1.21 × 10^−8^	1.1 × 10^−9^	2.10 × 10^−9^	2.2 × 10^−10^	2.03 × 10^−10^	2.1 × 10^−11^
9	1.23 × 10^−8^	1.1 × 10^−9^	2.07 × 10^−9^	2.0 × 10^−10^	2.54 × 10^−10^	2.4 × 10^−11^
10	1.17 × 10^−8^	1.3 × 10^−9^	1.73 × 10^−9^	1.5 × 10^−10^	2.31 × 10^−10^	3.1 × 10^−11^
12	1.28 × 10^−8^	1.2 × 10^−9^	1.82 × 10^−9^	1.7 × 10^−10^	1.88 × 10^−10^	2.4 × 10^−11^
15	1.29 × 10^−8^	1.2 × 10^−9^	1.66 × 10^−9^	1.6 × 10^−10^	2.21 × 10^−10^	2.3 × 10^−11^
20	1.13 × 10^−8^	1.0 × 10^−9^	1.74 × 10^−9^	1.5 × 10^−10^	2.40 × 10^−10^	2.5 × 10^−11^

**Table A5:** Mean escape time (τ) and associated errors of different number molecules confined in a 15-nm spherical cavity for different entrance–escape hole diameters (h) calculated by the interactive random walk models for 10^2^ runs.

Cavity Diameter = 15 nm								
N (Number of Molecules)	h = 1 nm		h = 2 nm		H = 5 nm		h = 7.5 nm	
	τ	Error (s)	τ (s)	Error (s)	τ (s)	Error (s)	τ (s)	Error (s)
1	1.41 × 10^−9^	1.4 × 10^−10^	6.17 × 10^−9^	7.0 × 10^−10^	7.61 × 10^−10^	1.1 × 10^−10^	2.95 × 10^−10^	3.6 × 10^−11^
2	1.38 × 10^−9^	1.4 × 10^−10^	7.26 × 10^−9^	8.3 × 10^−10^	8.49 × 10^−10^	8.1 × 10^−11^	2.88 × 10^−10^	2.7 × 10^−11^
3	1.57 × 10^−9^	1.6 × 10^−10^	6.39 × 10^−9^	7.5 × 10^−10^	7.52 × 10^−10^	7.7 × 10^−11^	3.88 × 10^−10^	5.9 × 10^−11^
4	1.50 × 10^−9^	1.4 × 10^−10^	7.29 × 10^−9^	6.8 × 10^−10^	8.47 × 10^−10^	8.8 × 10^−11^	3.93 × 10^−10^	5.2 × 10^−11^
5	1.39 × 10^−9^	1.4 × 10^−10^	7.09 × 10^−9^	6.8 × 10^−10^	8.47 × 10^−10^	9.2 × 10^−11^	3.77 × 10^−10^	4.3 × 10^−11^
6	1.46 × 10^−9^	1.3 × 10^−10^	6.13 × 10^−9^	7.7 × 10^−10^	8.37 × 10^−10^	1.1 × 10^−10^	3.21 × 10^−10^	4.2 × 10^−11^
7	1.27 × 10^−9^	1.2 × 10^−10^	7.26 × 10^−9^	7.4 × 10^−10^	6.41 × 10^−10^	7.0 × 10^−11^	3.72 × 10^−10^	4.3 × 10^−11^
8	1.30 × 10^−9^	1.4 × 10^−10^	6.37 × 10^−9^	6.8 × 10^−10^	6.60 × 10^−10^	6.8 × 10^−11^	2.65 × 10^−10^	3.3 × 10^−11^
9	1.82 × 10^−9^	1.8 × 10^−10^	6.11 × 10^−9^	6.1 × 10^−10^	7.77 × 10^−10^	7.6 × 10^−11^	3.88 × 10^−10^	4.1 × 10^−11^
10	1.41 × 10^−9^	1.4 × 10^−10^	6.49 × 10^−9^	6.9 × 10^−10^	8.88 × 10^−10^	1.0 × 10^−10^	2.67 × 10^−10^	3.3 × 10^−11^
12	1.23 × 10^−9^	1.5 × 10^−10^	6.90 × 10^−9^	7.8 × 10^−10^	7.38 × 10^−10^	8.1 × 10^−11^	3.21 × 10^−10^	4.0 × 10^−11^
15	1.53 × 10^−9^	1.5 × 10^−10^	6.52 × 10^−9^	6.9 × 10^−10^	7.58 × 10^−10^	7.7 × 10^−11^	2.96 × 10^−10^	3.2 × 10^−11^
20	1.47 × 10^−9^	1.3 × 10^−10^	6.19 × 10^−9^	5.3 × 10^−10^	9.07 × 10^−10^	8.9 × 10^−11^	3.39 × 10^−10^	4.5 × 10^−11^

**Table A6:** Mean escape time (τ) and associated errors of different number molecules confined in a 20-nm spherical cavity for different entrance–escape hole diameters (h) calculated by the interactive random walk models for 10^2^ runs.

Cavity Diameter = 20 nm								
N (Number of Molecules)	h = 1 nm		h = 2 nm		h = 5 nm		h = 10 nm	
	τ (s)	Error (s)	τ (s)	Error (s)	τ (s)	Error (s)	τ (s)	Error (s)
1	1.26 × 10^−7^	1.6 × 10^−8^	1.74 × 10^−8^	2.0 × 10^−9^	2.27 × 10^−9^	3.85 × 10^−10^	4.89 × 10^−10^	7.03 × 10^−11^
2	7.59 × 10^−8^	7.5 × 10^−9^	1.69 × 10^−8^	1.8 × 10^−9^	1.82 × 10^−9^	2.24 × 10^−10^	4.07 × 10^−10^	4.78 × 10^−11^
3	9.88 × 10^−8^	9.5 × 10^−9^	1.63 × 10^−8^	1.7 × 10^−9^	1.91 × 10^−9^	2.04 × 10^−10^	3.46 × 10^−10^	4.19 × 10^−11^
4	9.80 × 10^−8^	8.5 × 10^−9^	1.51 × 10^−8^	1.7 × 10^−9^	1.70 × 10^−9^	1.85 × 10^−10^	4.17 × 10^−10^	5.17 × 10^−11^
5	9.22 × 10^−8^	9.6 × 10^−9^	1.75 × 10^−8^	1.8 × 10^−9^	1.96 × 10^−9^	2.24 × 10^−10^	4.54 × 10^−10^	4.66 × 10^−11^
6	8.20 × 10^−8^	7.7 × 10^−9^	1.32 × 10^−8^	1.3 × 10^−9^	1.87 × 10^−9^	2.11 × 10^−10^	3.51 × 10^−10^	4.63 × 10^−11^
7	8.51 × 10^−8^	8.3 × 10^−9^	1.51 × 10^−8^	1.2 × 10^−9^	1.87 × 10^−9^	1.87 × 10^−10^	4.48 × 10^−10^	5.82 × 10^−11^
8	9.64 × 10^−8^	8.5 × 10^−9^	1.43 × 10^−8^	1.5 × 10^−9^	1.90 × 10^−9^	2.05 × 10^−10^	4.10 × 10^−10^	4.13 × 10^−11^
9	6.85 × 10^−8^	5.9 × 10^−9^	1.66 × 10^−8^	1.6 × 10^−9^	1.88 × 10^−9^	2.15 × 10^−10^	3.56 × 10^−10^	3.69 × 10^−11^
10	9.94 × 10^−8^	9.6 × 10^−9^	1.56 × 10^−8^	1.5 × 10^−9^	2.05 × 10^−9^	2.18 × 10^−10^	4.32 × 10^−10^	4.07 × 10^−11^
12	8.36 × 10^−8^	9.8 × 10^−9^	1.55 × 10^−8^	1.5 × 10^−9^	1.77 × 10^−9^	1.81 × 10^−10^	4.77 × 10^−10^	5.87 × 10^−11^
15	8.01 × 10^−8^	7.7 × 10^−9^	1.40 × 10^−8^	1.4 × 10^−9^	2.23 × 10^−9^	2.01 × 10^−10^	3.88 × 10^−10^	4.80 × 10^−11^
20	7.68 × 10^−8^	7.8 × 10^−9^	1.49 × 10^−8^	1.6 × 10^−9^	1.69 × 10^−9^	1.89 × 10^−10^	4.26 × 10^−10^	4.87 × 10^−11^
